# How do leaders' positive emotions improve employees’ psychological safety in China? The moderating effect of leader-member exchange

**DOI:** 10.1016/j.heliyon.2024.e25481

**Published:** 2024-01-30

**Authors:** Cong Wang, Jidong Yao, Lei Gao

**Affiliations:** a School of Economics and Management, Institute of Disaster Prevention, China; b Department of Economics and Management, Beijing City University, China

**Keywords:** Leader's positive emotion, psychological safety, Leader-member exchange, China

## Abstract

The extant research on psychological safety has explored mainly its consequences, with less attention paid to its formation mechanism and its applied situation. This study investigates the impact of leaders' positive emotions on employees' psychological safety based on a questionnaire survey of 319 employees working in 67 teams in China and verifies the moderating role of leader-member exchange in this relationship. According to the findings of the multilevel analysis, there is a positive association between leaders' positive emotions and employees' psychological safety, and leader-member exchange has a cross-level moderating influence on the link between leaders' positive emotions and subordinates’ psychological safety.

## 1 Introduction

Almost 60 % of the world population is in work.^1^ However, 15 % of working-age adults were estimated to have a mental disorder in 2019. Job insecurity and psychological insecurity pose a risk to mental health and productivity. Psychological safety refers to an employee's belief that even if he or she engages in a risky action, he or she will not be harmed [1]. Edmondson, an American management professor who was named the world's most influential management thinker in 2021, proposed in his monograph “The Fearless Organization” that organizations should create an environment in which employees can share their ideas, feedback, and constructive opinions without being ridiculed or punished. In such an environment, the company can thus have better ideas, better risk management, and more learning opportunities as well as make fewer catastrophic decisions. Extant studies have also confirmed the importance of psychological safety in organizational management, which has been confirmed that psychological safety can encourage employees to work better (including more work participation) and generate positive behavioral outcomes [2], including learning behavior [1,3], innovation [4], work performance [5], and voice behavior [6].

Previous studies have suggested that leadership behavior is one of the most important factors influencing the psychological safety of employees, whereas current leadership research focuses primarily on leadership characteristics and styles [7,8]. There is also a connection between leaders' emotions and leadership behavior. Van Kleef, Homan, and Cheshin (2012) demonstrated that an employee's emotional expression can influence the emotions, cognition, attitudes, and behaviors of others, a claim which also applies to leaders [9]. Studies have examined the role that psychological safety plays in mediating the link between emotions and employees' behavior at workplace. For instance, Liu et al. (2015) discovered that psychological safety mediates the association between employees' voice and their colleagues' moods [10]. However, previous research has rarely examined how and when leaders' emotions influence psychological safety in the Chinese cultural context. In Chinese enterprises with high guanxi, high power distance and a collectivist atmosphere, how do leaders' emotions affect employees' psychological safety? Can leaders' emotions stimulate employees' psychological safety? What are the boundary conditions that affect it? These are the questions that this study aims to further answer.

The daily emotional expression of leaders at the center of the organization impacts employees' emotions to some extent [11], which may affect employees' psychological safety. Emotion is one of the sources of motivation, according to psychological research. Emotion, on the other hand, has an infectious function. Emotions can affect and interact with one another, which may occur in the process of unconscious communication. Employees' emotions are affected to some extent by the daily emotional expression of leaders at the center of the organization [11], which may have an impact on employees' psychological safety. The Emotion as Social Information (EASI) model provides an effective theoretical framework for our research. According to EASI, when subordinates unconsciously “capture”, imitate, or consciously interpret the emotions expressed by leaders, their emotions, cognition, attitudes, behavior, and performance are affected [9]. For example, Liu et al. (2017) discovered that leaders' positive affect can induce their subordinates' positive affect through emotional infection, thereby improving subordinates’ psychological safety and voice behavior [6].

Simultaneously, employees' psychology and behavior are the result of the interactions between individual and organizational factors. The quality of the relationship between leaders and employees varies, and the impact of leaders' emotions on employees' psychological safety also varies. According to EASI, the relative predictive ability of reasoning and emotional responses depends on social relationship factors that include interpersonal relationships [12]. Leader-member exchange (LMX) is an important variable that reflects the leader-member relationship in the workplace, which serves as a representative resource and perceived organizational support. LMX can either strengthen or weaken the influence of other resource factors. Therefore, we used LMX to explore the role of different leader-member relationships in the relationship between leaders' positive emotions and employees’ psychological safety in this study.

To address these research gaps, this study conducts a multilevel analysis to investigate the relationship between leaders' positive emotions and employees' psychological safety and to test the moderating role of LMX in this relationship. Given the insufficiency of previous research to include leaders' emotional factors among the antecedents of psychological safety, the research on this topic could offer important information on how organizations and leadership styles induce psychological safety. This study can broaden the category of leadership factors that affect employees' psychological safety as well as the potential effects of leaders' positive emotions on employees’ behavior, thus enriching the literature on leader-employee relations.

## 2 Literature review and research assumptions

### 2.1 The concept and measurement of leaders’ emotions

Emotion is a fairly complicated psychological concept. Emotion refers to a relatively strong emotional experience triggered by a specific person or thing. A leader's emotion is defined in the organizational context as a leader's strong and transient adaptive response to an event, situation, person, or other entity, a definition that includes both the positive and negative emotions of leaders. With regard to positive emotions, pleasure, happiness, pride, enthusiasm, relief, optimism, affection, and power are most commonly reported [13]. Negative emotions include sadness, anger, fear, and so on.

According to the definition provided above, leaders' positive emotion refers to the positive emotional expression of leaders in the organization during interpersonal interactions. Since leaders are at the center of the organization, their daily emotional expression influences employees' emotions, which subsequently influence employees' behavior and performance [14,15]. Studies have also suggested that leaders can influence the emotions, motivations, and behaviors of subordinates through emotional expression [16,17]. Specifically, leaders' positive emotions are accompanied by trust between leaders and subordinates as well as by leaders' hopes and optimistic attitudes toward subordinates, which in turn affect subordinates’ attitudes and behaviors. Leaders exhibit humor, happiness, and relief, which allows them to interact more smoothly with their subordinates and generate less friction in daily interactions as well as to create a safe and friendly working atmosphere, thereby enabling subordinates to experience positive emotions [18] and create a safe psychological atmosphere.

According to EASI, leaders' emotions influence employees' emotions and work behavior via two distinct mechanisms, namely, affective reactions and inferential processes [12,19]. Affective reactions highlight the infectious function of leaders' emotional expression. Leaders' positive emotions can be transmitted to subordinates through emotional response pathways and thereby cause them to experience the same or similar emotional experiences [14]. For example, coaches' happy emotions can affect the happy emotions experienced by athletes both before and during competitions [20]. Inferential processes refer to situations in which employees make possible inferences regarding the information underlying the leader's emotional expression, which in turn affects employees' emotions and behaviors. When the leader is happy and relieved, for example, the employee may speculate that the leader is happy because his or her work performance meets the leader's expectations, so such an employee tends to feel uplifted and satisfied. The expression of gratitude by leaders can reduce subordinates' attribution of selfishness to leaders and produce positive work results [21]. The psychological capital of leaders is transmitted to the team through positive emotional expression, which can promote positive motivation within the team, thereby improving leadership effectiveness [22].

At present, the main methods for measuring leaders' emotions are the experimental method, the scale method, and the key event coding method. Through experimental techniques, the experimental method manipulates leaders' emotions [10]. To evaluate subjects' emotions and behaviors, such techniques focus on, for example, asking subjects to watch videos of leaders giving speeches, feedback, or task instructions or to report their psychological reactions and emotional states when they see leaders. The scale method allows subordinates to evaluate leaders' emotions over multiple periods by using the existing model's list of words describing emotions. For example, subordinates may be asked to evaluate the frequency of leaders' performance based on five discrete and discontinuous positive emotions (happiness, optimism, enthusiasm, passion, and interest) and five negative emotions of the same nature (disappointment, depression, anger, anxiety, and irritation) and to measure this factor repeatedly over a specific period [23]. The key event coding rule collects event experiences regarding leaders' emotions and behaviors through in-depth interviews, and the resulting data are analyzed thoroughly [12].

### 2.2 The concept and influencing factors of psychological safety

Schein and Bennis (1965) first proposed the concept of psychological safety in organizational change research [24]. Subsequently, Kahn (1990) and Edmondson (1999) conducted systematic research on the concept, function, and influencing factors of psychological safety [1,25]. The notion of psychological safety reveals the security needs of individuals in the organizational environment with the aim of reducing uncertainty and interpersonal risk, which are not only related to the work motivation and performance of individual employees but also affect the overall work efficiency and core competitiveness of the organization.

The concept of psychological safety can be defined at three levels: individual, team, and organization. Psychological safety at the individual level refers to the subjective belief that individuals can freely express their actual selves without worrying about adverse effects on their self-image, status, or career [25]. According to Kahn (1990), psychological safety is an important factor influencing employees' work engagement [25]. Employees' work engagement increases as their psychological safety increases. Psychological safety is defined as a shared belief on the part of team members that “taking risks is safe” at the team level. This notion is not the same as team cohesion; rather, it refers to a team atmosphere that represents the embodiment and aggregation of individual psychological safety [1]. Employees’ perceptions of the organizational atmosphere are referred to as psychological safety at the organizational level. Employees feel more psychologically safe in the organization if the organization allows them to try new methods to achieve goals, explains their roles more explicitly, and allows them to express their opinions freely at work [26].

Based on the definitions of these concepts, it is straightforward to conclude that psychological safety is a cognitive concept. It refers to a person's subjective perceptions of interpersonal relationships, team climate, and the organizational environment. It represents individuals' conviction that they do not have to fear experiencing negative treatment when they interact with others, express their opinions, and take on new responsibilities within the organization. The aforementioned activities in organizations are closely related to the participation of leaders. As a result, in studies about the factors influencing psychological safety, leaders' attitudes and behaviors have consistently been one of the most appealing factors. Previous research has found that whether employees feel psychologically safe is strongly influenced by their superiors [1,17,27]. For example, Edmondson (1999) and May et al. (2004) confirmed that supportive leadership characteristics can improve employees' team psychological safety [1,28]. Kanhn (1990) noted that a supportive, flexible, open, and clear management style can improve individual psychological safety [25]. Detert and Burris (2007) discovered that openness of management significantly explained the variation in team psychological safety [8] and that the inclusiveness of leadership could also significantly predict subordinates' team psychological safety [7]. Extant leadership studies have found that leadership characteristics and styles have positive impacts on promoting employees' psychological safety [[29], [30], [31], [32]].

However, according to the EASI model, in addition to leadership characteristics and styles, leaders' emotions can influence subordinates' emotions, attitudes, and behaviors through affective reactions and inferential processes. A large body of research has reported that when leaders exhibit positive emotions, subordinates are more likely to have positive emotional experiences [6,[33], [34], [35], [36]]. Leaders' positive emotions may also contribute to the formation of a positive team atmosphere [37,38]. Simultaneously, because the leader's emotions contain valuable information such as the leader's feelings at the time, social will, and expectations regarding the relationship between the two parties [39,40], subordinates interpret and judge the information contained in the leader's emotions [41], thereby forming a corresponding attitude [42]. Liu et al. (2017) discovered that leaders' emotions can improve subordinates' psychological safety [6]; that is, employees can express themselves freely during interactions with leaders [43,44].

Leaders typically control team resources and interaction patterns in organizations, thus allowing leaders to express their feelings more freely than subordinates. However, because of power and status differences, leaders' attitudes and emotions have a greater impact on their subordinates. Particularly in the context of Chinese culture, subordinates are more likely to pay attention to the emotional changes of leaders and confirm the emotional state of leaders by observing their words and expressions, which is influenced by high power distance^2^ and traditional Confucian ideology.^3^ Leaders' positive emotions can thus influence employees’ emotions and improve their psychological safety. Accordingly, Hypothesis 1 is proposed as follows.

H1 Leaders' positive emotions have a positive impact on subordinates' psychological safety.

### 2.3 The moderating role of LMX

Leader-member exchange (LMX) focuses on the quality of relationships between leaders and subordinates and how much influence leaders have over them. According to this theory, leaders employ various management techniques for various subordinates to create various kinds of exchange ties with subordinates due to time and resource limitations. Subordinates with higher quality LMX with leaders are referred to as “insiders”, and they receive more attention, opportunities, privileges, and so on from leaders. Other subordinates are referred to as “outsiders”, and their relationship with leaders is limited to the formal scope of authority [45]. Accordingly, different treatments of different subordinates may cause differences in their psychological feelings and thus affect employees’ feelings, work attitudes, and work performance [46,47].

In addition to its direct impact, the quality of LMX also indirectly affects the relationships among management style, emotion, and employee attitude and behavior of some leaders through mediation and moderation [48,49]. It is clear from the reasoning process associated with the EASI model alongside the meaning of LMX that LMX, as a social structural feature that can affect employees' cognition, impacts employees' behavioral evaluations of their superiors. According to the EASI model, the degree of information processing and the appropriateness of judgments made by observers can affect the intensity of observers’ affective reactions and inferential processes. Accordingly, researchers have emphasized “what affects the degree of information processing and appropriateness judgment of observers” when exploring the psychological mechanism underlying emotional effects [50,51].

The EASI model states that social relationship variables affect the relative predictability of cognitive and emotional reactions [12]. When leaders' feelings cause subordinates to feel inspired or threatened, subordinates with different LMX seek different additional information to explain the situation. Subordinates with a high LMX relationship perception look for more positive information to explain this situation based on the leaders' care and trust in everyday situations. Even their leaders' negative emotions tend to lead to more positive cognitions. Subordinates with low LMX relationship perception are more likely to gather external negative information to explain the current feelings of leaders and negative information erodes such subordinates' sense of self-worth and psychological safety. In “Guanxi” oriented and authority-oriented Chinese enterprises, employees are better at perceiving and experiencing leaders' emotions, which can affect employees' psychological safety. As long as employees confirm that they belong to the leader's “insiders”, they would have more positive evaluations of positive emotions of their leader, or understand that sometimes the venting of negative emotions may be the result of the stress of work and that their leaders require an outlet for emotional release. Then employees who possess such a cognition are more easily to prompt a sense of safety and able to recover in time even if they are temporarily depressed due to their leaders' emotions at that time and thus maintain a psychological sense of safety.

Accordingly, Hypothesis 2 is proposed as follows.

H2 LMX has a cross-level moderating effect on the relationship between leaders' positive emotions and subordinates' psychological safety.

**Fig. 1:**
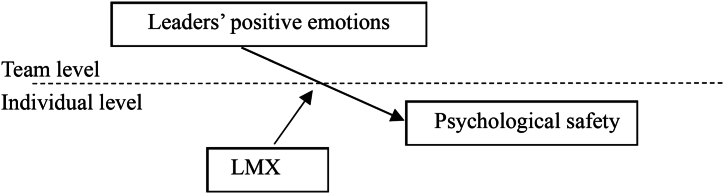
Conceptual model.

In summary, Fig. 1 depicts the research framework.

## 3 Method

### 3.1 Samples

Due to the need for team samples and considering feasibility, we have adopted convenient sampling. The questionnaire was distributed to 74 teams in Anhui Province, Hainan Province, and Beijing, representing a wide range of industries, including service and information technology. The three regions are located in middle, east and south of China, which to some extent ensures the representativeness. The data collection process was divided into three steps. First, contact the main team leader of the research enterprise to assure that person that all data are used only for research and do not include confidential industry and enterprise information. Second, explain in detail the purpose, steps, and precautions of the investigation and establish a time for the formal investigation. Teams were chosen from various occupational fields and divisions. With the approval of the research unit, the questionnaires used in this study were distributed to a total of 500 employees. Informed consent was obtained from all participants in the research. During the data collection process, we number the team, such as 1, 2, … And then further number the members within the team, such as 1–2, 1–2, … In this way, each team and its members can be distinguished. Team research aggregation criteria was strictly followed in the study, with each team consisting of at least three members. Teams from which fewer than three employee questionnaires were collected were excluded. After excluding some responses because of missing data and poor quality, this study ultimately included 67 teams featuring a total of 319 employees.

Among the leaders in the effective sample, the mean number of employees under their direction ranged from 3 to 8, with a mean value of 4.76. Teams with three members accounted for 38.81 % of the total, teams with four members accounted for 22.39 %, and teams with five members accounted for 10.45 %. Samples are presented in Table 1.

**Table 1:** Samples.

Items	Variable	Frequency	Percent (%)
Gender	Male	137	42.9
	Female	182	57.1
Age	Younger than 25	49	15.4
	25–35	111	34.8
	36–45	89	27.9
	Older than 46	70	21.9
Level of education	Below college	77	24.1
	Associate degree	75	23.5
	Bachelor's degree	140	43.9
	Master's degree or above	27	8.5
Working experience	1–3 years	99	31.0
	4–10 years	94	29.5
	11–25 years	84	26.3
	25 years or more	42	13.2

### 3.2 Measures

The questionnaire includes measurement items for the three research variables, leaders' positive emotions, LMX, and psychological safety, as well as control variables. In line with research on leaders' positive emotions [23], this study asked subordinates to report the frequency with which their supervisors displayed the following five positive emotions (i.e., happy, enthusiastic, optimistic, excited, and funny) on a scale featuring typical questions such as “Does your leader display the following emotions at work”. A 5-point Likert scale was used, with 1 indicating rarely and 5 indicating very often. The Cronbach's alpha value of the scale was 0.93.

The psychological safety questionnaire was based on a five-item scale developed by May, Gilson, and Harter (2004) and revised by Li and Yan (2007) [28], which included typical questions such as “What I express at work is my true opinion”. A 5-point Likert scale was used, with 1 indicating strong disagreement and 5 indicating strong agreement. The Cronbach's alpha value of the scale was 0.86.

LMX was conducted using Graen and Uhl-Bien's (1995) 7-item scale, which featured typical questions such as “My leader is very aware of my difficulties and needs at work”. A 5-point Likert scale was used, with 1 indicating strongly disagree and 5 indicating strongly agree [45]. The Cronbach's alpha value of this scale was 0.92.

Cronbach's α of the three research variables are both greater than 0.80, indicating good internal consistency among various measurement items, and the scales have high reliability.

Control variables included the demographic characteristics of the respondents. The codes used were as follows: gender (0 = male, 1 = female), age (1 = under 25, 2 = 25–35, 3 = 36–45, 4 = over 46), level of education (1 = below college, 2 = associate degree, 3 = bachelors' degree, 4 = master's degree or above), and working experience (1 = 1–3 years, 2 = 4–10 years, 3 = 11–25 years, 4 = 25 years or more) were coded.

### 3.3 Analytical strategy

The theoretical model developed in this study includes both individual-level and team-level variables, with the team-level variable being leaders' positive emotions; accordingly, the direct consensus approach was used to integrate the team-level data. Before data integration, the study examined the three most commonly used indicators in multilevel analysis as proposed by Bliese (2000) [52], namely, within-group consistency γ_wg_, within-group correlation ICC (1), and ICC (2). The results showed that for leaders' positive emotions, γ_wg_ values across teams ranged from 0.81 to 0.99, with median and mean values of 0.94 and 0.91, respectively, ICC (1) = 0.34, ICC (2) = 0.71, and F (66, 252) = 4.41, p < 0. 01, thus indicating that data integration was appropriate. As a result, with regard to data statistics, this study employed a cross-level analysis method. The specific data analysis was divided into three parts. The first section used descriptive statistics and correlation analysis to describe the basic characteristics of all variables as well as the correlations among variables; the second section conducted validity tests to confirm that the scale and variables were valid; and the third section featured a multilevel regression analysis to test the influence of leaders' positive emotions on employees’ psychological safety as well as the moderating role of LMX on this connection.

## 4 Results

### 4.1 Descriptives and correlations

The study's hypotheses received some preliminary support from the means, standard deviations, and correlation coefficients among the variables. Table 2 shows that at the individual level, leaders' positive emotions were positively correlated with psychological safety (r = 0.52, *p* < 0. 01) and LMX (r = 0.73, *p* < 0. 01). The correlation coefficients between variables are all moderately correlated, indicating the possibility of verifying hypotheses.

**Table 2:** Means, standard deviations, and correlation coefficients among variables.

	M	SD	1	2	3	4	5	6
1. Gender	0.57	0.50	1					
2. Age	2.56	1.00	−0.07	1				
3. Education	2.37	0.94	0.00	0.04	1			
4. Working experience with leaders	2.22	1.03	0.00	0.73**	0.13*	1		
5. Leaders' positive emotions (team level)	3.86	0.79	−0.09	−0.04	−0.13*	−0.14*	1	
6. Psychological safety	3.97	0.66	−0.11	0.00	−0.27**	−0.03	0.52**	
7. LMX	3.93	0.67	−0.18**	0.01	−0.17**	−0.07	0.73**	0.65**

Note: *N* = 319, *p < 0.05, **p < 0.01.

### 4.2 Validity tests

Utilizing factor analysis, the scale's construct validity was tested.

First, exploratory factor analysis (EFA) was conducted to test validity. The software calculated the results of the KMO test and Bartlett's sphericity test, which revealed that the KMOs of leaders' positive emotions, psychological safety, and LMX were all greater than 0.7. The significance levels for the three variables in Bartlett's sphericity test were much less than 0.01, indicating that factor analysis was appropriate. Principal component analysis (maximum variance method) was used to assess the validity of the items in this study. The loadings matrix of the rotated factors was used to examine the variable factor loadings. The factor analysis for the items measuring leaders' positive emotions revealed that one factor could be extracted from five items with a cumulative contribution of variance of 77.859 %, the factor analysis for the items measuring psychological safety revealed that one factor could be extracted from five items with a cumulative contribution of variance of 64.810 %, and the factor analysis for the items measuring LMX revealed that one factor could be extracted from seven items with a cumulative contribution of variance of 66.840 %. The factor loading of each item of these three scales was greater than 0.7, thus indicating a high degree of explanatory power.

Second, confirmatory factor analysis (CFA) was conducted to test the model's convergent validity and discriminant validity. The AVE values of the model, the composite reliability (CR), the square root values of AVE, and the correlation coefficients are presented in Table 3, and HTMT (heterotrait-monotrait ratio) values are presented in Table 4. The AVE values corresponding to a total of three variables are all greater than 0.5, and the CR values are all higher than 0.7. The square root value of AVE of leaders' positive emotions, psychological safety, and LMX are all greater than the maximum value of the correlation coefficients among factors, i.e., 0.851 > 0.729, 0.750 > 0.653, and 0.784 > 0.729 respectively. HTMT values are all lower than 0.85. Results of the CFA thus indicate that the three variables exhibit good convergent and discriminant validity.

**Table 3:** Tests of convergent and discriminant validity.

	AVE	CR	Leaders' positive emotions	Psychological safety	LMX
Leaders' positive emotions	0.724	0.929	0.851		
Psychological safety	0.563	0.864	0.515	0.75	
LMX	0.614	0.917	0.729	0.653	0.784

χ2 = 354.783，df = 116，*р*<0.01，NFI = 0.912，NNFI = 0.928，CFI = 0.939，RMSEA = 0.08，SRMR = 0.042.

**Table 4:** HTMT.

	Leaders' positive emotion	Psychological safety	LMX
Leaders' positive emotions	–		
Psychological safety	0.572	–	
LMX	0.791	0.737	–

### 4.3 Common method variance

Although only employees provided the data for this study, we were able to significantly control for common method variation by using procedural controls (Podsakoff et al., 2012) throughout the research procedure [53]. In addition, this study applied the Harmon's one-way test, which is commonly used in academia, to test for common method bias in results. The principal component analysis extracted three factors and obtained an unrotated first factor that explained 25.65 % of the covariance, which is much less than the reference value of 40 %, thus indicating that no single factor explains most of the variance.

### 4.4 Hypothesis testing

The results of the test of each hypothesis (Table 5-a, Table 5-b) are as follows. Hypothesis 1 proposed that leaders' positive emotions have a positive impact on subordinates' psychological safety. According to the Model 1 data presented in the table, leaders' positive emotions were positively correlated with employees’ psychological safety (β = 0.49, *p* < 0.01); hence, Hypothesis 1 is supported.

**Table 5-a:** Regression analysis for leaders’ positive emotions predicting psychological safety.

Variables	Psychological safety							
	Model 1			Model 2			Collinearity statistics	
Individual level	β	t value	Sig	β	t value	Sig	Tolerance	VIF
Intercept	2.51	8.51	.00	1.58	6.27	.00		
Gender	−0.09	−1.32	.19	0.00	.056	.96	.96	1.04
Age	0.03	.64	.52	−0.03	−.68	.50	.44	2.27
Education	−0.13	−3.81	.00	−0.11	−3.66	.00	.94	1.07
Working experience with leaders	0.03	.68	.50	0.06	1.54	.12	.45	2.21
LMX				0.57	11.065	.00	.63	1.59
Team level								
Leaders' positive emotions	0.49	8.39	.00	0.11	1.844	.07	.63	1.60

**Table 5-b:** Regression analysis for leaders’ positive emotions predicting psychological safety.

Variables	Psychological safety			LMX		
	Model 3			Model 4		
Individual level	β	t value	Sig	β	t value	Sig
Intercept	1.45	5.68	.00	1.62	5.53	.00
Gender	−.00	−.03	.97	−0.16	−2.57	.01
Age	−.02	−.56	.58	0.10	2.30	.02
Education	−.10	−3.22	.00	−0.04	−1.31	.19
Working experience with leaders	.07	1.67	.10	−0.05	−1.18	.24
LMX	0.57	11.086	.00			
Team level						
Leaders' positive emotions	0.13	2.204	.03	0.66	12.20	.00
Interactions						
Leaders' positive emotions × LMX	0.18	2.546	.01			

As Table 5–a has shown, tolerances of both independent variables are greater than 0.6, and VIFs are lower than 2, which indicates that there is basically no multicollinearity problem in this model.

Hypothesis 2 showed that LMX had a cross-level moderating effect on the relationship between leaders' positive emotions and subordinates' psychological safety. This study tested Hypothesis 2 using hierarchical regression analysis. In the process of this hierarchical regression analysis, we took subordinates' psychological safety as the dependent variable, and the steps to put its predictive variables into the regression analysis were (1) control variables; (2) two main effects (leaders' positive emotions and LMX); and (3) the product of leaders' positive emotions and LMX. Moreover, we employed centered regression model when testing the moderating effect to avoid multicollinearity.

Model 2 in Table 5–a showed a significant positive relationship between LMX and psychological safety (β = 0.57, *p* < 0.01), Model 4 in Table 5–b showed a significant positive relationship between leaders' positive emotions and LMX (β = 0.66, *p* < 0.01), and Model 3 in Table 5–b showed that the product of leaders' positive emotions and LMX were positively related to subordinates' psychological safety (β = 0.18, *p* < 0. 05). As a result, Hypothesis 2 was supported, which states that LMX moderates the impact of leaders' positive emotions on their subordinates' psychological safety.

The regression analysis of the hypothesis test is summarized in Table 6.

**Table 6:** Summary of regression analysis.

Variables	Psychological safety			LMX
Individual level	Model 1	Model 2	Model 3	Model 4
Intercept	2.51**	1.58**	1.45**	1.62**
Gender	−0.09	0.00	−.00	−0.16*
Age	0.03	−0.03	−.02	0.10*
Education	−0.13**	−0.11**	−.10**	−0.04
Working experience with leaders	0.03	0.06	.07	−0.05
LMX		0.57**	0.57**	
Team level				
Leaders' positive emotions	0.49**	0.11	0.13*	0.66**
Interactions				
Leaders' positive emotions × LMX			0.18*	

Note: *N* = 319，*p < 0.05，**p < 0.01.

**Fig. 2:**
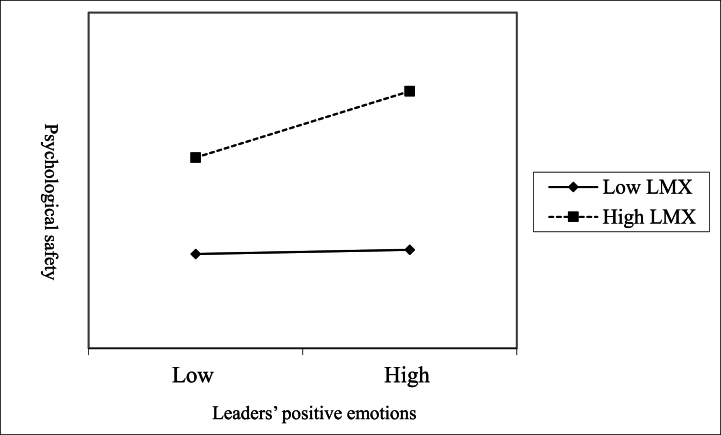
The moderating effect of LMX on the relationship between leaders' positive emotions and psychological safety.

Furthermore, the present study developed a schematic representation of this moderating effect (see Fig. 2) [54], which shows that when LMX took two different conditional values (i.e., the mean plus one standard deviation and the mean minus one standard deviation), in teams with strong LMX, leaders' positive emotions had a significant positive effect on subordinates' psychological safety (β = 0.25, *p* < 0.01), and in teams with weak leader-member exchange, leaders' positive emotions had no significant effect on subordinates’ psychological safety (β = 0.01, n. s.), thereby providing further support for Hypothesis 2.

## 5 Discussion and conclusion

The results of the present study show leaders' emotions as important social signals could impact employees' daily affective reactions [6,19]. Specifically, leaders' positive emotions are positively associated with employees’ psychological safety. This finding further validates the mechanism of affective reactions and the inferential processes proposed by the EASI model, according to which leaders appropriate and proper expressions of their positive emotions can be transmitted directly to employees, not only infecting their emotions and instilling in them a positive and proactive mindset but also giving them a perception of a secure and reassuring psychological experience.

The study also confirmed that LMX acts as a moderator in the relationship between leaders' positive emotions and employees' psychological safety. Employees who have high-quality LMX relationships perceive their leaders to be caring, accommodating, and supportive, resulting in a stronger sense of psychological safety and more pronounced experiences and feedback regarding the expression of leaders' positive emotions. These perceptions are extremely beneficial for developing employees’ self-confidence and creating a relaxed and harmonious corporate environment. Employees in such an environment are not ostracized for being their true selves at work, and they are thus more willing to express their true thoughts while stimulating their creativity and initiative to offer higher performance to the organization.

### 5.1 Theoretical implications

Our findings contribute to the literature on leaders’ emotions and leadership in three ways.

First, a theoretical model of the mechanism underlying the effect of leaders' emotions on subordinates' psychology from an emotional perspective was conducted and validated. Most previous studies on the antecedents of psychological safety have focused on leadership behavior (such as empowering leadership) [30,31] and management styles (conflict management) [25]. The results of this study indicate that leaders' positive emotions are an important factor in the improvement of employees' psychological safety, which broadens the range of leadership factors that affect employees' psychological safety. The EASI model is also used to explore the possible consequences of leaders' positive emotions on employees’ behavior.

Second, in theoretical terms, the understanding of the influencing process of leaders' positive emotions was enhanced. This study integrated emotional contagion and signaling mechanisms to explain why leaders' positive emotions influence followers' psychological safety. Given that the EASI model focuses on how observers process other people's emotions, EASI was employed to explain the mechanism underlying the impact of leaders' positive emotions on subordinates' psychology in the Chinese context.

Third, the influence of the quality of leader-employee relationships on the ways in which employees respond to leaders' emotions was examined and the boundary conditions for the psychological effects of leaders' positive emotions on employees were tested. The study contributes to EASI theory by offering proof that emotional reasoning processes are not always in operation in this context due to the regulatory role of conditional factors. As a variable associated with the organizational climate, leader-employee relationships can effectively explain why different employees exhibit different psychological states in response to positive emotions from leaders. The concept of LMX is helpful for clarifying the boundary conditions under which leaders’ positive emotions exert their influence, and it enriches the theoretical applications of leader-member exchange as a boundary mechanism.

### 5.2 Practical implications

This study for management implies that leaders need to manage their own emotions well in their management practices, focus on the positive effects of positive emotions on employees, and be wary of negative and undesirable emotions. Although maintaining positive emotions in daily management is difficult for leaders, it is a necessary skill for them. Subordinates are more sensitive to their leaders' emotional expressions, especially in the unique Chinese cultural context. The phrases that parents frequently use when teaching their children who are just beginning to enter the workplace, such as “see the wind and set the helm”, “observe somebody's words and gestures”, and “see what's going on”, highlight how important it is for Chinese subordinates to understand their leaders' emotions. As a result, leaders should be mindful of when and where they express their emotions in their daily work and avoid expressing negative emotions of their personal lives or other work matters to their subordinates. Even if subordinates' performance is not satisfactory, it is necessary to discuss the facts, avoid expressing anger via personal attacks, and adjust the communication style to suit the personality characteristics of the subordinates to reduce psychological insecurity among employees, increase trust in leadership, and thus motivate employees to act favorably toward the organization. China is characterized by an oriental culture that advocates implicit and introverted attitudes. Traditional social education pays little attention to the cultivation of skills related to emotional expression, especially the emotional expression of positive emotions such as affection. Managers should pay attention to the need to understand the input mechanism associated with emotional information in sufficient depth while strengthening their attention to the output of emotional information, starting with the cultivation of their emotional expression ability, to further improve the accuracy of individual emotional expression.

Simultaneously, high-quality LMX can help employees obtain psychological safety, alleviate their negative emotions, and enable them to participate in more difficult tasks and take more risks. High-quality LMX relationships may promote employees' work motivation and behavior more than the organization's rules and regulations, especially in the “guanxi”-focused organizational environment in China. Therefore, leaders should communicate with employees in a timely manner, express concern for them, listen to their opinions, resolve their demands, and establish high-quality LMX relationships in a fair manner with employees.

### 5.3 Limitations and future research

Although this study filled some gaps in the extant research, it also faces certain limitations. First, this study did not take into account leaders' negative emotions. Positive emotions exhibit lower intensity and less persistence than negative emotions [55]. As a result, future research can assess the impact of both positive and negative emotions on employees' psychological safety. Second, this study's research methodology is cross-sectional, which may impact the persuasiveness of the causal explanations proposed by the research model. It would be preferable to collect longitudinal data over a longer period in future research to conduct a more rigorous test of the research questions. The data referenced in this study are based primarily on employee self-reports. Although the Harmon's one-factor test results show that common method bias did not affect the study's conclusions, the diversity of data sources should be considered in future research to ensure more accurate and scientific data collection. Finally, the research sample comes from the Chinese cultural context, but there may be more specific regional cultural differences among employees. Therefore, future research should incorporate the examination of cultural differences among individual employees to obtain more general conclusions and apply them to a larger practical scope.

## Data availability statement

Data will be made available on request.

## CRediT authorship contribution statement

**Cong Wang:** Writing – review & editing, Writing – original draft, Methodology, Investigation, Funding acquisition, Formal analysis, Conceptualization. **Jidong Yao:** Writing – review & editing, Writing – original draft, Methodology, Conceptualization. **Lei Gao:** Writing – review & editing, Data curation, Conceptualization.

## Declaration of competing interest

The authors declare that they have no known competing financial interests or personal relationships that could have appeared to influence the work reported in this paper.

## Footnotes

1 World employment and social outlook - Trends 2022. Geneva: International Labour Organization; 2022 (https://www.ilo.org/global/research/global-reports/weso/trends2022/WCMS_834081/lang--en/index.htm, accessed 26 August 2022).

2 In Hofstede's (2005) cross-cultural country study, China is a country with high power distance, which means that due to institutional reasons, China places more emphasis on the binding force of power. People with lower status in Chinese society have a higher acceptance of the unequal distribution of power in society or organizations.

3 As the mainstream ideology in China, Confucianism emphasizes the hierarchical order, advocating for the principle of ‘the monarch guide the minister’. It believes that the governance of a country depends on the stability of the hierarchical order.
